# Expression and release of glucose-regulated protein-78 (GRP78) in multiple myeloma

**DOI:** 10.18632/oncotarget.17353

**Published:** 2017-04-21

**Authors:** Normann Steiner, Bojana Borjan, Roman Hajek, Karin Jöhrer, Georg Göbel, Wolfgang Willenbacher, Johann Kern, Eberhard Gunsilius, Gerold Untergasser

**Affiliations:** ^1^ Department of Internal Medicine V, Hematology and Medical Oncology, Innsbruck Medical University, Innsbruck, Austria; ^2^ Laboratory for Tumor Biology and Angiogenesis, Innsbruck Medical University, Innsbruck, Austria; ^3^ Tyrolean Cancer Research Institute, Innsbruck, Austria; ^4^ Department of Medical Statistics, Informatics and Health Economics, Innsbruck Medical University, Innsbruck, Austria; ^5^ Faculty of Medicine, University of Ostrava, Ostrava, Czech Republic; ^6^ Department of Hemato-Oncology, University Hospital Ostrava, Ostrava, Czech Republic

**Keywords:** multiple myeloma, MGUS, GRP78, ELISA, prognostic marker

## Abstract

**Introduction:**

Multiple myeloma (MM) is a plasma cell neoplasm that is mostly incurable due to acquired resistance during the treatment course. Thus, we evaluated expression and release of glucose-regulated protein 78 kDa (GRP78/BiP), an endoplasmic reticulum (ER) based pro-survival chaperone involved in immunoglobulin folding and unfolded protein responses.

**Results:**

GRP78 protein expression in the ER and on the cell surface did not significantly differ between MGUS, NDMM and RRMM patients although there was a trend to higher surface expression in RRMM. In bone marrow plasma, the amount of released GRP78 protein was not significantly increased between MGUS-, NDMM- and RRMM patients. MM cells of the three cell lines release GRP78 as full-length protein under apoptotic, but not under acidotic or ER-stress conditions. In necrosis, only proteolytic fragments of GRP78 were detected in supernatants of MM cells.

**Materials and Methods:**

GRP78 protein expression and plasma levels were quantified in bone marrow aspirates of patients with monoclonal gammopathy of undetermined significance (MGUS, *n* = 29), newly diagnosed MM (NDMM, *n* = 29) and with relapsed/refractory MM (RRMM, *n* = 15) by immunohistochemistry and sandwich ELISA. The human MM cell lines U266, NCI-H929 and OPM-2 were used for functional GRP78 release- and processing studies after induction of acidosis, ER stress, apoptosis and necrosis.

**Conclusions:**

Ectopic expression of GRP78 on cell membrane or its release in the microenvironment is not a suitable marker to distinguish MGUS from NDMM and RRMM.

## INTRODUCTION

Multiple myeloma (MM) remains incurable despite the implementation of panoply of novel drugs such as thalidomide, lenalidomide, bortezomib or carfilzomib into the therapeutic algorithms. Although the overall and progression free survival has improved considerably due to novel treatment options [1–3], resistance to anti-myeloma drugs occurs [4]. Moreover, highly heterogeneous genetic alterations in MM cells and the respective bone marrow microenvironment are responsible for clinical manifestations of MM and play a central role for resistance to treatments [5]. Hitherto, no reliable biomarkers predicting response to treatment are available.

Glucose-regulated protein 78 kDa (GRP78), also referred to as BiP (heavy chain immunoglobulin binding protein), is the major endoplasmic reticulum (ER) chaperone regulating ER stress signaling processes. GRP78 has been described in different intracellular compartments of malignant cells, e.g. in the ER or the mitochondria, and even on the cell surface [6]. This key chaperone protein of the unfolded protein response (UPR) has been shown by us to be responsible for resistance to the anti-angiogenic activity of the proteasome-inhibitor bortezomib and, by other groups, to BRAF-inhibitors [6–10]. Increased expression of GRP78 protein was associated with poor response to treatment in prostate, stomach, colon, lung, ovary and breast cancer [9–13]. Until now, GRP78 expression has never been explored as a predictive biomarker in multiple myeloma, although an ectopic expression of GRP78 on the cell membrane has been reported in a subgroup of MM patients [14]. Recently, a human immunoglobulin-M antibody (PAT-SM6) has been described to recognize GRP78 on cell membrane and to induce apoptosis in MM cells [14].

There is no descriptive and functional data on protein levels of GRP78 in the bone marrow of patients with plasma cell disorders, such as monoclonal gammopathy of undetermined significance (MGUS, a premalignant condition ultimately leading to MM), newly diagnosed MM (NDMM) and relapsed/refractory MM (RRMM). Therefore, we studied GRP78 expression, translocation to cell membrane, release and its processing in MM cells undergoing apoptosis, necrosis, acidosis or ER-stress. We validated a sandwich ELISA system by native eukaryotic protein for the quantification of GRP78 and its respective fragments and determined GRP78 levels in plasma of bone marrow samples deriving from MGUS, NDMM and RRMM patients.

## RESULTS

### Expression of GRP78 in the endoplasmic reticulum (ER) and on the cell surface of plasma cells of patients with MGUS, NDMM and RRMM

**Figure 1:**
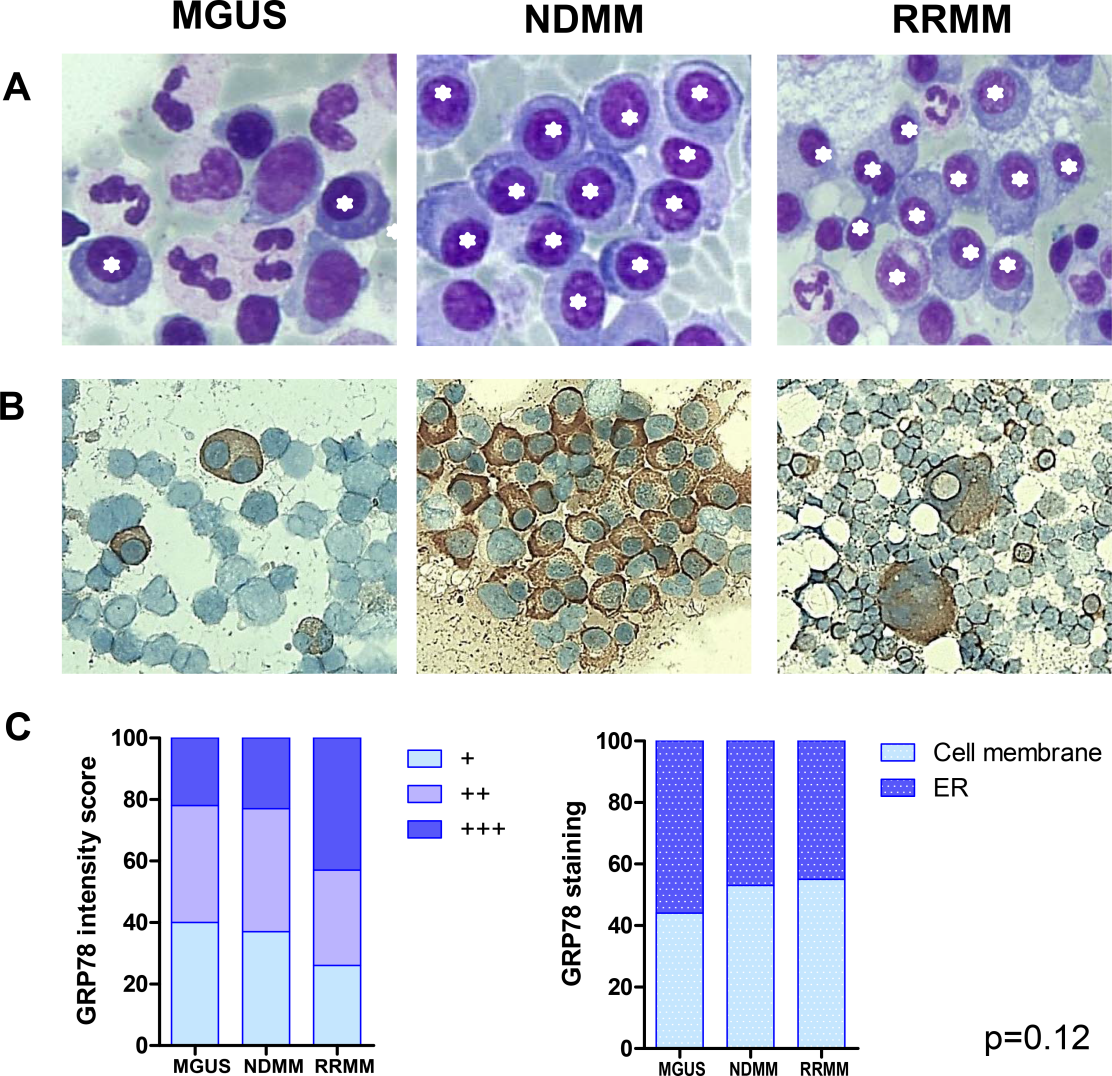
GRP78 expression in endoplasmic reticulum of plasma cells of patients with MGUS, NDMM and RRMM (**A**) Plasma cells were visualized by stereo microscope after Giemsa staining of bone marrow aspirates. Asterisks mark plasma cells. (**B**) Immunohistochemistry (IHC) with GRP78 antibody was performed in formalin-fixed bone marrow *smears* in patients with MGUS, NDMM and RRMM. No significant differences in GRP78 staining intensity and localization of GRP78 could be observed between plasma cells in MGUS, NDMM and RRMM patients. (**C**) Statistical analysis of GRP78 expression in patients with MGUS, NDMM and RRMM after IHC on air-dried, formalin-fixed bone marrow *smears*. Mean staining intensity/plasma cell. No significant differences in GRP78 staining intensity and localization of GRP78 could be observed between plasma cells in MGUS, NDMM and RRMM patients (*p* = 0.12).

GRP78 has been reported as the key regulator of ER-stress signaling processes, which are responsible for cell survival, adaptation and ER homeostasis. MM cells, due to their production of dysfunctional immunoglobulins resulting in the intracellular accumulation of huge amounts of unfolded protein, suffer considerable “ER-stress”. Thus, we analyzed GRP78 protein expression in the ER of bone-marrow derived MM cells of patients with MGUS, NDMM, and RRMM, visualizing it by stereo microscopy and immunochemistry. As expected, in MGUS a low frequency of plasma cells in the bone-marrow in comparison to NDMM and RRMM was found (Figure 1A). RRMM samples showed a higher GRP78 staining intensity (+++ vs. +/++) on the cell membrane than in the ER compared to MGUS and NDMM samples but no significant differences in GRP78 staining intensity and localization were found (Figure 1B and Figure 1C, *p* = 0.12).

### Validation of an ELISA for the quantification of human GRP78

Since we did not observe significant differences in the cellular expression levels of MM cells in regard to GRP78 we were interested to study release of GRP78 under pathological conditions or high ER stress.

**Figure 2:**
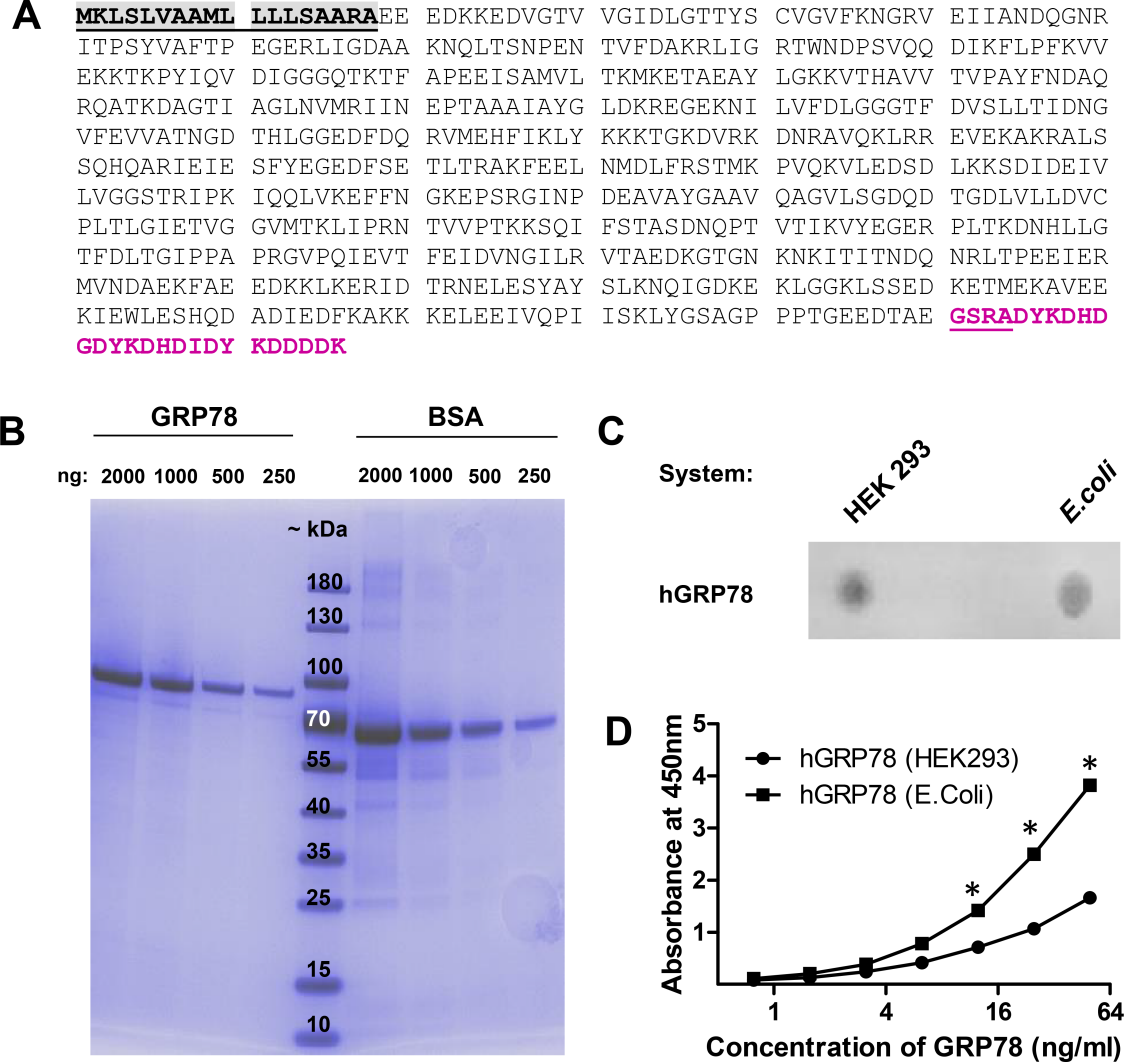
Validation of the human GRP78 ELISA with eukaryotic sGRP78-FLAG as standard for measurement (**A**) Amino acid sequence of human GRP78 (BiP) fused to a c-terminal FLAG tag for affinity purification from supernatants of human cells. Grey = signal peptide, purple = FLAG tag. (**B**) Affinity purification of FLAG tagged human GRP78 from the supernatant of transiently transfected HEK293 cells. Quantity and quality of GRP78 was visualized with a reference BSA standard and molecular weight marker by a 4–20% gradient SDS-Page and Page blue staining. GRP78-FLAG has an estimated size of 78 kDa. (**C**) The polyclonal sheep-anti human GRP78 detection antibody recognizes in Dot Blot analysis both isoforms of GRP78, those produced in human cells (HEK293) and that produced in prokaryotes (*E.-coli*). (**D**) Human plasma, negative for GRP78, was spiked with different amounts of native eukaryotic GRP78-FLAG and reduced prokaryotic GRP78 protein standard and measured in the sandwich ELISA. ELISA detects prokaryotic-folded GRP78 with a higher affinity than the eukaryotic (ER-folded and modified) protein from human cells. * indicate *p* < 0.05.

Unfortunately, no validated and specific-monoclonal antibodies for the quantification of native human GRP78 were available. Thus, we tested commercially available GRP78 ELISA systems for the specific measurement of human GRP78 and found one system (Biovendor) that specifically quantifies hGRP78 in plasma samples. This system is based on a prokaryotic hGRP78 as a standard and a polyclonal sheep antiserum as coating and detection antibody. However, human proteins expressed in bacteria often exhibit distorted folding and thus fail to reach their native form and function. To ensure proper protein folding and glycosylation in the endoplasmic reticulum, we generated a recombinant hGRP78 standard in the human HEK293FT cell line (sequence in Figure 2A). Purified protein was quantified before usage in the ELISA by the external protein standard BSA (Figure 2B). Since the polyclonal sheep antiserum was developed against hGRP78, produced and purified from *E.-coli*, we tested it for its interaction with native, ER-folded and glycosylated hGRP78 that resembles GRP78 in human plasma cells. The polyclonal sheep antiserum specifically detects hGRP78 produced in HEK293FT cells, as determined by Dot Blot analysis in direct comparison to hGRP78 generated in *E.-coli* (Figure 2C). Notably, this antiserum displays a substantially higher binding affinity to GRP78, when applied in a sandwich ELISA system setting. This ER-folded protein purified from human cancer cell supernatants under native conditions, and thus resembling tumor-derived GRP78, was detected with a significant lower affinity when spiked into a negative sample in the respective biological matrix (Figure 2D).

### MM cells release full length GRP78 under apoptosis, and proteolytic fragments under necrosis

**Figure 3:**
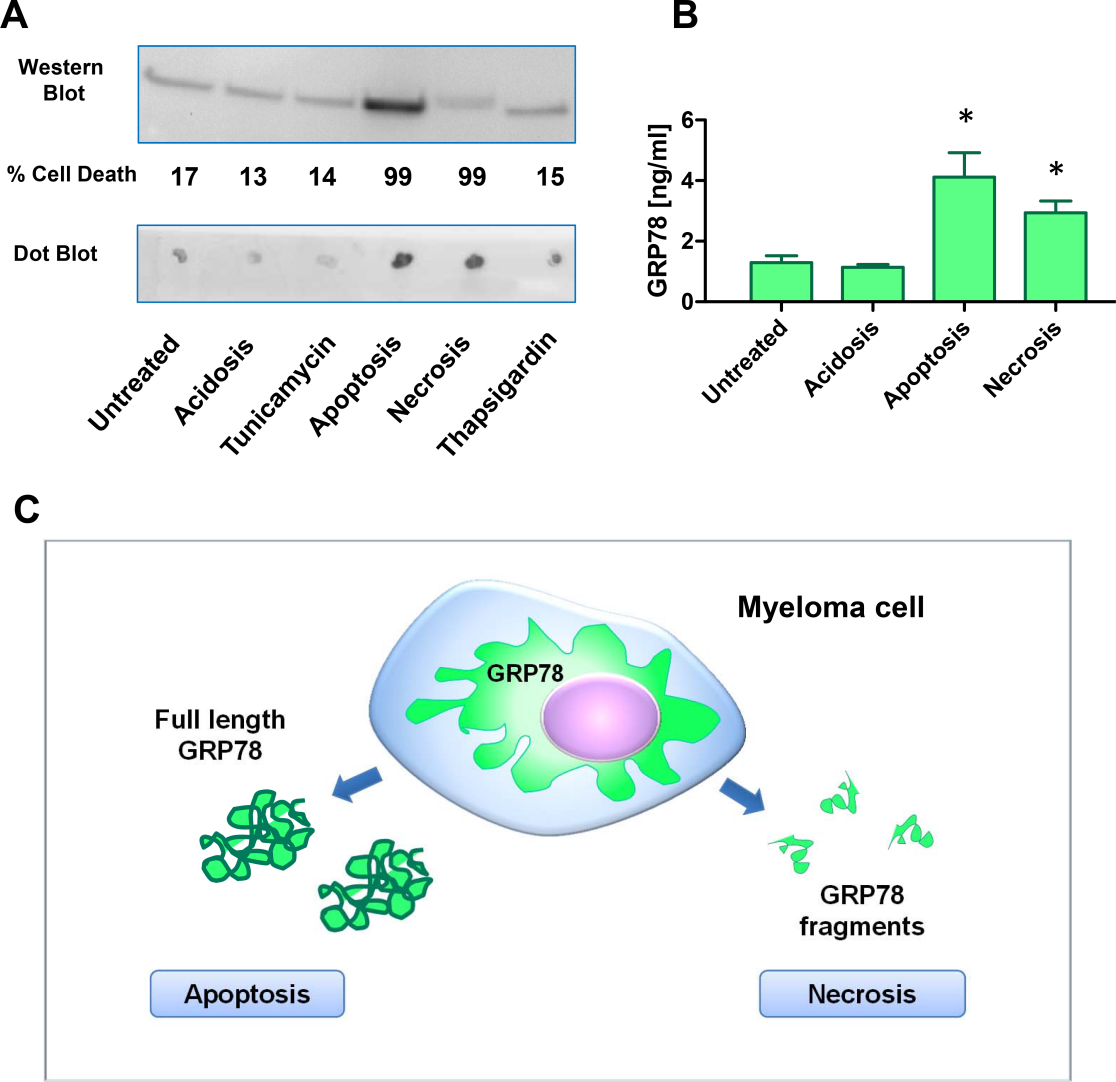
Plasma cells undergoing apoptosis or necrosis release full length or cleaved fragments of GRP78 (**A**) Acidosis (pH 6.3), apoptosis, necrosis, and ER-stress were induced in NCI-H929 multiple myeloma cell line by treatment with acetic acid, 100 μM calcimycin, 100 mM H2O2, 30ng/ml tunicamycin, and 1nM thapsigargin for 72 h, respectively. Supernatants were analyzed by the sheep-anti human GRP78 polyclonal antiserum used in the sandwich ELISA. Apoptosis resulted in an increase of full length GRP78 in the Western Blot analysis. Necrosis gave no increased signal in Western Blot analysis, but on Dot Blot analysis immune-reactive GRP78 fragments increased significantly. (**B**) Supernatants of NCI-H929 cells under acidosis (pH 6.3), apoptosis and necrosis were measured in the GRP78 sandwich ELISA. Apoptosis as well as necrosis gave significant higher signals for immune-reactive GRP78 in the supernatants. * indicate *p* < 0.05 (**C**) Hypothetical model of GRP78 release and processing of MM cells under apoptotic and necrotic conditions. ELISA will measure apoptosis as well as necrosis of MM cells in bone marrow plasma samples.

Under normal acidotic, and ER-stress culture conditions (thapsigargin, tunicamycin), MM cells released very low amounts of full length hGRP78 protein, whereas under apoptotic conditions, a significantly higher release of full length hGRP78 protein was found (Figure 3A). There was no release of full length hGRP78 protein after necrosis, but an increase of GRP78 immuno-reactive protein fragments. GRP78 protein fragments were detected in cells undergoing necrosis by Dot Blot analysis, whereas Western Blot did not display the protein with molecular weight at 78 kDa. Apoptotic cells (A23187) were discriminated from necrotic cells (H_2_O_2_) by measuring kinetics of cell death, loss of membrane integrity and exposure of phosphatidylserin with 7AAD and Annexin-V. Data were confirmed by ELISA, where we found an increase of immune-reactive GRP78 protein or its fragments in apoptosis and necrosis (Figure 3B). Of note, discrimination between full length protein and processed fragments of GRP78, identified in the bone-marrow plasma of MM patients by GRP78-ELISA, was not possible. Therefore, GRP78 signals can originate from apoptotic or necrotic MM cells of the bone marrow (Figure 3C).

### Bone-marrow and peripheral blood plasma levels of GRP78 do not significantly differ between MGUS, NDMM and RRMM patients

Myeloma cells can upregulate production and release of GRP78 upon stress conditions, such as disturbance of ER homeostasis, e.g. due to exposure to therapeutic antimyeloma agents [10]. Moreover, MM cells could adapt to permanent ER-stress due to antibody production by increasing chaperone production, such as GRP78. Thus, we wanted to know whether RRMM patients have higher GRP78 levels in bone-marrow plasma than NDMM or MGUS patients. As shown in Table 1, GRP78 protein expression did not significantly differ between MGUS, NDMM and RRMM, respectively (2.5 ng/mL [median; range 1.7–6.4] vs. 3.1 ng/mL [median; range 1.8–7.3] and 4.0 ng/mL [median; range 1.6–7.8], *p* = 0.8). Moreover, we measured GRP78 levels also in peripheral blood samples to find a correlation to pathological processes in the bone marrow reflected by peripheral plasma. GRP78 expression in peripheral blood (healthy donors, MGUS-, NDMM-, and RRMM patients) was marginally lower than in bone marrow aspirates of the different cohorts. Similar ranges of GRP78 protein expression were also observed in peripheral blood of our three cohorts, not differing from samples of healthy volunteers (Supplementary Table 1).

**Table 1:** GRP78 expression levels in bone marrow increased from MGUS to NDMM and RRMM

Patients	GRP78 levels			
	median	[95% CI]	IQR	*p-*value
**MGUS (*n* = 29)**	**2.5 ng/mL**	1.7–6.4	1.6–8.4	
**NDMM pts (*n* = 29)**	**3.1 ng/mL**	1.8–7.3	1.7–11.4	
**RRMM pts. (*n* = 15)**	**4.0 ng/mL**	1.6–7.8	1.6–7.3	
**All pts (73)**	**3.3 ng/mL**	2.0–5.4	1.6–8.4	*p* = 0.8

Abbrevations: MGUS, monoclonal gammopathy undetermined significance; MM, multiple myeloma; NDMM, newly diagnosed multiple myeloma; RRMM, relapsed refractory multiple myeloma; [95% CI], Confidence interval; IQR, Interquartile range.

### Correlation of GRP78 levels in bone marrow plasma with clinical parameters in patients with MGUS, NDMM and RRMM

GRP78 protein levels of MGUS, NDMM and RRMM patients were correlated with prognostic parameters, such as the type of the clonal immunoglobulin, β-2 microglobulin, lactate-dehydrogenase, creatinine, serum calcium, hemoglobin, osteolytic bone lesions and cytogenetic risk constellation (Table 2A and 2B). In patients with myeloma bone disease, i.e. osteolytic bone lesions, we found a trend to higher GRP78 protein levels in RRMM patients compared to NDMM patients (median 4.0 ng/mL vs. 2.7 ng/mL). In both, the RRMM and NDMM patients, concentrations of GRP78 were marginally higher in patients with cytogenetic defined high-risk disease (i.e. del17p13, t(4;14), t(14;16) and 1q21 gain) compared to patients with standard risk (median 5.8 ng/mL and 5.4 ng/mL vs. 2.8 ng/ml and 2.3 ng/mL). Interestingly, RRMM and NDMM patients with a higher percentage of bone marrow plasma cells displayed decreased GRP78 protein levels compared to those with a lower percentage. GRP78 protein expression was higher in plasma of bone marrow blood samples of RRMM patients treated with 4th and more therapy lines when compared to RRMM patients treated with 2nd/3rd therapy lines (median 5.4 ng/mL vs. 2.9 ng /mL). RRMM patients treated with either proteasome inhibitors (PI) or immunomodulatory agents (IMiDs) displayed equal concentrations of GRP78 protein in plasma of bone marrow blood samples (median 4.0 ng/mL vs. 4.3 ng/mL). However, most likely due to the limited patient number (especially patients with MGUS rarely receive bone-marrow examination) these data reached not statistical significance (*p* > 0.05).

**Table 2A:** Correlation of GRP78 protein with clinical parameters

	MGUS			NDMM			RRMM		
	GRP78 levels (ng/mL)			GRP78 levels (ng/mL)			GRP78 levels (ng/mL)		
Parameter	*N*(%)	median	[95% CI]	*N*(%)	median	[95%CI]	*N*(%)	median	[95% CI]
Patients	29 (100)	2.5	1.7–6.4	29 (100)	3.1	1.8–7.3	15 (100)	4.0	1.7–7.7
Age < median	16 (55)	3.1	1.2–8.4	7 (24)	1.8	1.5–15.0	13 (87)	3.3	1.6–6.8
Age > median	13 (45)	2.3	1.6–6.4	22 (76)	3.6	2.1–7.3	2 (13)	6.6	5.4–7.8
Sex f/m									
f	11 (38)	2.3	1.0–14.4	16 (55)	5.7	1.8–15.0	9 (60)	6.8	1.8–10.5
m	18 (62)	3.1	1.6–8.1	13 (45)	2.3	1.5–6.2	6 (40)	1.6	1.5–5.4
Type of Ig heavy chain (serum)									
IgG	18 (62)	2.1	1.4–5.2	15 (52)	2.3	1.5–10.5	7 (47)	1.8	1.6–6.8
IgM	7 (24)	5.8	1.6–55.7	0	0		2 (13)	21.4	1.2–41.8
IgA	2 (7)	56.4	1.2–111.6	6 (21)	3.5	1.8–12.4	1 (7)	Not av.	
IgD	0			1 (3)	Not av.		0		
Light chain only	2 (7)	4.8	1.7–7.8	7 (24)	7.3	1.8–12.0	5 (33)	4.8	3.3–8.7
Type of Ig light chain (serum)									
Kappa	17 (59)	3.6	1.6–15.7	17 (59)	2.7	1.5–6.0	7 (47)	5.4	1.5–21.5
Lambda	12 (41)	2.4	1.2–7.1	12 (41)	9.3	2.0–21.4	8 (53)	3.6	1.7–8.7
β-2 microglobulin > UNV	10 (35)	7.1	1.7–53.6	23 (79)	2.7	1.8–6.8	10 (67)	3.3	1.5–6.8
LDH > UNV	1 (3)			6 (21)	4.2	1.8–82.8	5 (33)	5.4	3.3–8.7
Creatinine ≥ 1.3 mg/dl	5 (17)	6.4	0.5–111.6	17 (59)	2.7	1.7–15.0	6 (40)	4.4	1.6–6.6
Serum calcium > UNV	1 (3)	Not av.		4 (14)	6.6	1.4–95.2	3 (20)	8.7	3.3–10.5
Haemoglobin ≤ 12 g/dl	14 (48)	3.0	1.7–14.4	25 (86)	3.1	1.8–7.4	9 (60)	4.8	1.5–7.3
Platelets <1 00,000/mm^3^	1 (3)	Not av.		6 (21)	4.3	1.7–20.0	8 (53)	5.1	1.5–8.7

Abbrevations: N, number of patients; Ig, Immunoglobulin; UNV, upper normal value; LDH, lactate-dehydrogenase.

**Table 2B:** Correlation of GRP78 protein with clinical parameter

	MGUS			NDMM			RRMM		
	GRP78 levels (ng/mL)			GRP78 levels (ng/mL)			GRP78 levels (ng/mL)		
Parameter	*N* (%)	median	[95%CI]	*N* (%)	median	[95%CI]	*N* (%)	median	[95%CI]
Patients	29 (100)	2.5	1.7-6.4	29 (100)	3.1	1.8-7.3	15 (100)	4.0	1.7-7.7
Osteolytic bone lesions	0			27 (93)	2.7	1.8-7.4	15(100)	4.0	1.7-7.8
Cytogenetic standard risk	1 (3)			9 (31)	2.3	1.7-16.5	4(26)	2.8	1.2-5.4
Cytogenetic high risk	1 (3)			15 (52)	5.4	1.5-13.2	10(67)	5.8	1.7-8.7
Cytogenetic not avail.	27(94)			5 (17)			1(7)	Not av.	
BMPCs < 10% BMPCs < 20% BMPCs 20 - < 50% BMPCs >= 50% BMPCs percentage not avail.	29 (100) 0 0 0 0	2.5	1.7-6.4	0 7(24) 6 (21) 7 (24) 9 (31)	4.2 3.4 2.3	1.8-19.4 1.0-63.0 1.5.-48.5	0 2 (14) 5 (33) 3 (20) 5 (33)	5.1 4.0 1.5	1.6-8.7 1.8-6.8 1.2-10.5
Therapy lines at samples collection									
1^st^ line therapy	0			0			0		
2^nd^ line + 3^rd^ line therapy	0			0			8 (53)	2.9	1.6-7.8
4^th^ and higher lines of therapy	0			0			7 (47)	5.4	1.5-21.5
BTZ based therapy at sample collection	0			0			9 (60)	4.0	1.6-7.7
IMiDs based therapy at sample collection	0			0			6 (40)	4.3	1.6-25.0

Abbrevations: BMPCs, Bone marrow plasma cells; IMiD, Immunomodulatory drugs; BTZ, Bortezomib.

## DISCUSSION

In this study, GRP78 protein expression and levels in plasma of bone marrow blood samples were studied in patients with different stages of myeloma disease (MGUS, NDMM, RRMM) to evaluate GRP78 as a marker to discriminate plasma cell disorders, i.e. stratifying for patients having MM cell clones with high resistance to ER-stress and proteasome inhibition. To our knowledge, this is the first study analyzing GRP78 expression levels and GRP78 release into the microenvironment in primary bone-marrow samples of MGUS, NDMM and RRMM patients.

Plasma cells need an enhanced ER activity to ensure their high protein synthesis activity, thus resulting in an activation of the UPR. This is even more the case in malignant plasma cells which produce huge amounts of aberrant immunoglobulins. Stress-inducible ER chaperone proteins, such as GRP78, glucose-regulated protein 94 (GRP94), calreticulin (CRT) and protein disulfide isomerase (PDI), facilitate protein folding [15]. Especially, GRP78 can translocate to the cell surface contributing to cell survival, proliferation, metastasis and angiogenesis [6] [9–13]. GRP78 was identified by our group as being responsible for resistance to the antiangiogenic activity of the proteasome inhibitor bortezomib, which is a key drug for the treatment of myeloma patients [10]. In analogy to other ER proteins containing KDEL retention signal, GRP78 can be translocated to the plasma membrane and released into the extracellular space upon disturbance of the ER calcium pool due to stress or saturation of the KDEL receptors [16]. When used as antitumor vaccines, GRP94 can induce antitumor immune responses and protein disulfide isomerase (PDI) shows pro-survival activity [15]. Depending on the length and intensity of ER stress, UPR signaling can lead either to an activation of pro-survival mechanisms or to apoptosis in tumor cells [15].

Here, we validated an ELISA for human GRP78 for the measurement of plasma samples and MM supernatants. Myeloma cells release very low amounts of full length hGRP78 under normal acidotic and ER-stress culture conditions. A significantly higher release of full length hGRP78 was observed under apoptotic conditions. These findings are new, since ER stress did not induce higher GRP78 expression in myeloma cells and GRP78 release in apoptosis has never been described before. By Dot Blot analysis, increased GRP78 immuno-reactive protein fragments were detected in cells undergoing necrosis. However, in our GRP78-ELISA, discrimination between full length protein and processed fragments of GRP78 in plasma of MM patients was not possible. Hitherto, it is known that bortezomib treatment leads to GRP78 co-localization with aggresomes, activates GRP78-dependent autophagy and improves anti-myeloma effect [17]. Of course, at present it is unclear whether cleaved fragments have similar effects on the microenvironment as described for GRP78 [18]. It is feasible that they might also activate immune cells and modulate immune responses in the tumor. Moreover, GRP78 signals can originate from all cells of the bone marrow when apoptotic or necrotic, not necessarily and exclusive from plasma cells. In our immunohistochemical analysis of bone marrow smears, strong expression of GRP78 protein was found in MGUS-, NDMM-, and RRMM- patients. Our results are in line with the study of Rasche et al. where GRP78 expression increased with disease progression and therapy with monoclonal antibody PAT-SM6 was explored in one MM patient with increased GRP78 and drug-resistance (bortezomib based- and IMiD based therapy) [19], but major differences on protein expression and intensity of GRP78 staining were not observed in bone marrow smears of our three cohorts.

Our results confirm that GRP78 is also expressed in bone-marrow of MGUS patients. MGUS is a premalignant disorder with a clonal plasma cell count in the bone marrow of < 10% and an annual risk of progression to MM in about 1% [20]. Reasons for a trend to lower levels of GRP78 in bone marrow samples of MGUS patients compared to the NDMM and RRMM cohort could be the generally lower plasma cell infiltration in bone marrow and therefore, knowing that GRP78 is a stress-inducible chaperone, the smaller proportion of driver clones for transformation in symptomatic MM.

We expected that RRMM patients, especially patients pretreated with bortezomib, have higher levels of intracellular GRP78 than untreated NDMM and MGUS patients [17, 21]. Regarding our study, differences of RRMM compared to MGUS- and NDMM patients showed merely a statistical trend. We assume, that in comparison to normal plasma cells MM cells of RRMM and of NDMM patients are more “stressed” due to the high production of misfolded antibodies.

However, increased plasma levels of GRP78 most likely originate from dead cells which were proposed as the main source of GRP78 in the preclinical part of this study. Further experimental studies are needed to elucidate this hypothesis.

GRP78 protein expression of bone marrow aspirates was also correlated with its expression in peripheral blood samples (healthy donors, MGUS-, NDMM-, and RRMM patients), as very likely the quality of the aspirate impacts on bone marrow levels of GRP78. In peripheral blood plasma GRP78 expression was marginally lower than in bone marrow aspirates of the different cohorts. Considering the limited number of samples GRP78 protein levels in peripheral blood of our three cohorts (MGUS, NDMM, and RRMM), correlated well with bone marrow plasma levels.

We noted that surgical processes in the bone have a high impact on elevated GRP78 levels, due to generation of apoptotic or necrotic cell death and that aspirates by needle biopsy are not as dramatically influenced by cell death due to surgery.

From the clinical perspective, GRP78 protein levels in plasma of bone marrow do not differ between primary and refractory MM and GRP78 cannot be used as biomarker to monitor disease progression or destruction of the bone microenvironment by MM cells. Nevertheless, future prospective studies will examine whether GRP78 increases during treatment or relapse of single patients and if changes in GRP78 are indicative for development of resistance in single patients.

## MATERIALS AND METHODS

### Ethics statement

Investigations have been conducted in accordance with the ethical standards and according to the Declaration of Helsinki and according to national and international guidelines and have been approved by the authors’ institutional review board (AN2015-0034 346/4.13; AN5064 Innsbruck) and (20/1/2011; Brno).

### Patients

Patients with MGUS (*n* = 29), newly diagnosed MM (NDMM; *n* = 29) and relapsed/refractory MM (RRMM; *n* = 15) according to the International Myeloma Working Group (IMWG) criteria [22], were included in the study population (Table 3). Bone marrow aspirates of all 73 patients were analyzed. Bone marrow smears were prepared on glass coverslips and dried and stored until further analysis. Bone marrow samples underwent centrifugation for 10 min at 1000 × *g* and obtained bone marrow plasma was collected and stored at −80°C.

**Table 3:** Patient demographics and characteristics (*n* = 73)

Parameter	MGUS		NDMM		RRMM	
	*n = 29*	%	*n* = 29	%	*n* = 15	%
Median age (range), years	64 (59–75)		72 (65–79)		59 (50–63)	
Sex f/m						
f	11	38	16	55	9	60
m	18	62	13	45	6	40
ISS						
I			7	24	5	33
II			6	21	5	33
III			16	55	5	33
Type of Ig heavy chain (serum)						
IgG	18	62	15	52	7	47
IgM	7	24	0	0	2	13
IgA	2	7	6	21	1	7
IgD	0	0	1	3	0	0
Light chain only	2	7	7	24	5	33
Type of Ig light chain (serum)						
Kappa	17	59	17	59	7	47
Lambda	12	41	12	41	8	53
β-2 microglobulin >UNV	10	35	23	86	10	71
LDH >UNV	1	3	6	21	5	33
Creatinine ≥1.3 mg/dl	5	17	17	59	6	40
Serum calcium > UNV	1	3	4	14	3	20
Haemoglobin ≤ 12 g/dl	14	48	25	86	9	60
Platelets < 100,000/mm^3^	1	3	6	21	8	53
Osteolytic bone lesions	0	0	27	93	15	100
Cytogenetic standard risk	1	3	9	31	4	27
Cytogenetic high risk	1	3	15	52	10	66
Cytogenetic not available	27	94	5	17	1	7
Therapy lines at samples collection						
1st line					0	0
2nd line					3	20
3rd line					5	33
4th line					1	7
5th line					1	7
6th line					4	26
7th line					1	7
BTZ based therapy					9	60
IMiD based therapy					6	40

Abbrevations: N, number of patients; ISS, International staging system; Ig, Immunoglobulin; UNV, upper normal value; LDH, lactate-dehydrogenase; IMiD, Immunomodulatory drugs; BTZ, Bortezomib.

### Substances

A-23187 Free Acid (Calcimycin) was purchased from Molecular Probes^™^ and dissolved in DMSO (SIGMA Biochemicals) to a stock solution of 10 mM. Hydrogen peroxide (H_2_O_2_) solution 30% was purchased from Merck and acetic acid from Carl Roth. Protein Standard (analytical standard 200 mg/ml [BSA]) was obtained from SIGMA Biochemicals. Thapsigargin was purchased from Molecular Probes, Tunicamycin was obtained from Sigma Aldrich and Bortezomib from Selleckchem.

### Cell culture

The MM cell line NCI-H929 (ACC 163) was purchased from DSMZ Germany, U266 and OPM-2 were purchased from ATCC. All used MM cell lines were authenticated by us by flow cytometry (CD138+/CD38+) and STR-profiling. Cells were grown in the recommended cell media (RPMI 1640; Lonza) supplemented with 10% fetal calf serum, 105 IU/L penicillin, 100 mg/L streptomycin, and 2 mmol/L glutamine in the presence of 5% CO_2_. For GRP78 release/processing studies MM cells were cultivated in Protein-Free Hybridoma medium (PFHM-II, Thermo Fisher Scientific).

### Western/dot blot analysis

Conditioned supernatants of multiple myeloma NCI-H929 cells were obtained by washing the cells in PBS and culturing them in Protein-Free Hybridoma medium for 72 h. Supernatants were collected and centrifuged at 12,000 × *g* for 30 min to remove cell debris. They were further concentrated using Amicon Ultra-0.5 centrifugal filter unit with ultracel-10 membrane (UFC501096, Milipore). For dot blot assay, 1 μl aliquots of 5 x concentrated samples were spotted on nitrocellulose membranes (Protean BA 85, GE Healthcare Life Sciences, Whatman). Released proteins were denatured, separated with 4–20% SDS-PAGE (Criterion TGX, Bio-Rad) and transferred to an Immuno-Blot TM polyvinylidene difluoride (PVDF) membrane (Bio-Rad) in western blot analysis. After blocking the membrane in 3% BSA dissolved in TBS, membranes were incubated 2 h at room temperature in 3% BSA with a primary polyclonal antibody GRP78 supplied with the GRP78-ELISA Kit (Biovendor). Afterwards, membranes were incubated with an anti-biotin HRP conjugated antibody (CST) diluted 1:1000. After washing, a chemiluminescent substrate (LumiGLO Reagent and Peroxide, Cell Signaling Technology) was added to the membrane, which was then exposed in the Chemidoc XRS station (Bio-Rad Laboratories).

### Flow cytometry

Cell death was evaluated using human FITC-labeled Annexin V (EnzoTM) and 7-amino-actinomycin D (7-AAD, Beckman Coulter) staining. Therefore, cells were resuspended in 200 μL Annexin V Binding Buffer (BD Biosciences) with 2 μL Annexin-V and 2 μL PI (20 μg/mL), incubated for 15 min, washed and resuspended in PBS/5% FCS prior to analysis. Cells were examined in a FACS Calibur (Becton-Dickinson, Heidelberg, Germany). We cultivated human MM cell lines in serum free medium and induced acidosis (pH 6.3), apoptosis (A-23817, calcimycin) or necrosis (H_2_O_2_) or sublethal ER stress (Thapsigargin, Tunicamycin, Bortezomib). Apoptosis and necrosis were confirmed by flow cytometric analysis of nuclear 7AAD uptake and/or Annexin-V binding to the cell membrane. Apoptotic MM cells (Annexin-V positive, 7-AAD negative) and necrotic cells (Annexin-V and 7-AAD positive) were measured 24 h after stimulation.

### Generation of a recombinant eukaryotic human sGRP78-FLAG

The HSPA5 cDNA (BC020235, GE-Dharmacon) was subcloned into the p3x FLAG CMV 14 expression vector (SIGMA Biochemicals) by the use of primers, amplifying the full length open reading frame without the c-terminal KDEL retention signal (GRP78-for: 5-GATATCGCAGGCTCCACCATG; GRP78-rev: 5-ggatccttgcagataattgg), and the KOD polymerase (Calbiochem). PCR products were purified (PCR-Wizard, Promega) and cloned into the expression vector using Eco-RV, Xba-I and the Quick Ligase Kit (all NEB). Thereafter, ligated constructs were transformed into chemo competent Top10 cells (Invitrogen), and propagated for large scale production. Plasmids were purified by the Midi-Prep Kit (Qiagen) and sequenced for correct fusion to the C-Terminal FLAG tag (see: Supplementary Table 1). Plasmids were transfected into HEK293FT (Invitrogen) cells using Lipofectamin 2000 (Invitrogen). For recombinant protein production, transfected HEK293FT cells were cultivated in serum-free hybridoma medium (Thermo Fisher Scientific) for 4 days. Following centrifugation of supernatants of HEK293FT at the speed of 10,000 × g for 20 min at 4°C., recombinant FLAG-fusion protein was purified by affinity chromatography and FLAG-M2 agarose beads (SIGMA Biochemicals). The protein was eluted from column under native conditions by an excess of 3 × FLAG peptide (SIGMA Biochemicals). Thereafter, protein was dialyzed against PBS (Fresenius) and purity was analyzed by SDS-PAGE with Page Blue Protein staining (Thermo Scientific).

### GRP78 sandwich ELISA

To determine the GRP78 levels, a commercially available Human Glucose-Regulated Protein 78 ELISA (BioVendor) was used according to the manufacturer's instructions. Briefly, plasma samples, diluted 3x with the assay buffer, were incubated in duplicates in the ELISA plate, pre-coated with capture anti-GRP78 antibody, for 2 h at room temperature. Three washes were performed to remove unbound proteins and each well was incubated with a biotin labeled anti-GRP78 detection antibody at room temperature for 1 h. After repeated washing steps, the plate was treated with a streptavidin-horseradish peroxidase conjugate. Following an enzymatic reaction with the substrate for peroxidase (room temperature, 10 min) and subsequent termination of the color development, the absorbance at 450 nm and 630 nm was measured using a microplate ELISA reader.

### Immunohistochemistry

Bone marrow smears were air-dried and fixed in cold methanol: acetone - 1:1 (v/v) for 10 min on ice and subsequently permeabilized by incubating the slides in 0.2% Triton X-100 in PBS for 20 min on ice. Permeabilization was followed by incubation with Dako blocking buffer which contained 10% FCS for 60 min at room temperature. Slides were incubated overnight in a humidified chamber at 4°C with primary anti-human antibodies (rabbit anti-human GRP78 from Acris at 1:150 dilution in Dako buffer/10% FCS). After rinsing in PBS, cells were incubated with biotinylated goat anti-rabbit IgG secondary antibody (Vector Laboratories) at a dilution 1:100 for 60 min at room temperature. Following washing step in PBS, slides were incubated with Vectastain Elite ABC Reagent, immunoperoxidase detection system containing Avidin DH and biotinylated peroxidase H, for 45 min at RT. Thereafter, slides were incubated in peroxidase substrate solution, DAB dection kit, for 5 min. Counterstaining was carried out with Meyers hemalum for 20 s. Slides were finally washed with tap water and distilled water and mounted in aquatex. Imaging was conducted with spinning disc confocal microscopic system (Ultra VIEW VoX; Perkin Elmer, Waltham, MA, USA) that was connected to a Zeiss Axio Observer Z1 inverted microscope (Zeiss). Images were acquired with Velocity software (Perkin Elmer) using a 63x oil immersion objective with a numerical aperture of 1.42. Scoring of immunostaining results was performed by two hematologists (NS and WW) blinded to all clinicopathological data. Intensity of GRP78 staining was scored with +, ++ or +++.

### Statistics

Statistical evaluation was performed using SPSS statistical software (version 20.0; SPSS Inc., Chicago, IL, USA). All tests for statistical significance were two-sided. The unpaired *t*-test and Kruskal-Wallis test were used to identify differences between the three groups. Wilcoxon test, Pearson's correlation and analyses were performed using GraphPad Prism TM6 (GraphPad Software Inc., La Jolla, CA, USA). A *p*-value of < 0.05 was considered as statistically significant.

## SUPPLEMENTARY MATERIALS TABLES


