# Supplementary material for: Expression and release of glucose-regulated protein-78 (GRP78) in multiple myeloma
Source: Oncotarget. 2017 Apr 21;8(34):56243–54. doi: 10.18632/oncotarget.17353 (PMC5593558; doi:10.18632/oncotarget.17353)
Supplement: Supplementary file 1 [file oncotarget-08-56243-s001.pdf]

Expression and release of glucose-regulated protein-78 (GRP78) in multiple myeloma

Supplementary Materials

Supplementary Table 1: GRP78 expression levels in peripheral blood (PB)

Patients	GRP78 levels			
	median	[95% CI]	IQR	*p*-value
Control P. (*n*=6)	4.0 ng/mL	0.6–35	0.7–27.5	
MGUS (*n* = 5)	3.6 ng/mL	0.3–50	1.2–50	
NDMM pts (*n* = 6)	1.3 ng/mL	0.2–39.1	0.2–14.3	
RRMM pts. (*n* = 6)	1.6 ng/mL	0.9–6.3	1.2–4.3	
All pts (23)	2.0 ng/mL	0.7–4.5	0.7–10.3	$p = 0.4$

Control *P* = control person, MGUS = monoclonal gammopathy undetermined significance, MM = multiple myeloma, NDMM = newly diagnosed multiple myeloma, RRMM = relapsed refractory multiple myeloma, IQR = Interquartile range.
